# Supplementary material for: Global soil profiles indicate depth-dependent soil carbon losses under a warmer climate
Source: Nat Commun. 2022 Sep 20;13:5514. doi: 10.1038/s41467-022-33278-w (PMC9489695; doi:10.1038/s41467-022-33278-w)
Supplement: Supplementary file 1 — Supplementary Information [file 41467_2022_33278_MOESM1_ESM.pdf]

Supplementary Information for

Global soil profiles indicate depth-dependent soil carbon losses under a warmer climate

Mingming Wang et al.

Supplementary Table 1. The effects of biome type, soil type and landform on the response of soil organic carbon stock (SOC_s) to warming estimated by meta-analysis. Q_M, the heterogeneity caused by the relevant factors (i.e., biome type, soil type, landform and precipitation seasonality), and *p* values show the statistical significance level of the difference.

**Warming level (°C)**	**Soil depth (m)**	**Sample size (*n*)**	**Biome type**		**Soil type**		**Landform**		**Precipitation seasonality**	
			Q_M	*p*	Q_M	*p*	Q_M	*p*	Q_M	*p*
**1**	0–0.3	7319	16.4	0.06	8.53	0.67	1.4	0.5	1.96	0.37
	0.3–1	6112	6.89	0.55	8.27	0.69	1.28	0.53	2.88	0.24
	1–2	3205	13.55	0.09	4.87	0.94	1.6	0.45	2.8	0.25
**2**	0–0.3	6090	32.67	<0.01	14.58	0.2	2.33	0.31	4.23	0.12
	0.3–1	5076	11.74	0.16	14.55	0.2	1.04	0.59	6.31	0.04
	1–2	2519	14.33	0.07	8.91	0.63	3.9	0.14	0.27	0.87
**3**	0–0.3	5346	34.46	0	17.31	0.1	3.28	0.19	9.83	0.01
	0.3–1	4436	10.77	0.22	11.24	0.42	3.58	0.17	23.62	<0.01
	1–2	2152	10.06	0.26	6.26	0.86	2.14	0.34	2.5	0.29
**4**	0–0.3	4765	31.52	<0.01	14.6	0.2	5.39	0.07	13.73	<0.01
	0.3–1	3885	9.64	0.29	8.61	0.66	2.99	0.22	5.1	0.08
	1–2	1809	20.15	0.01	17.07	0.11	2.56	0.28	2.95	0.23
**5**	0–0.3	4339	43.75	<0.01	21.55	0.03	12.46	<0.01	4.77	0.09
	0.3–1	3531	5.59	0.69	7.82	0.73	12.28	<0.01	0.29	0.87
	1–2	1649	9.44	0.31	12.29	0.34	6.02	0.05	1.11	0.57

Supplementary Table 2. The effects of biome type, soil type and landform on the response of soil organic carbon content (SOC_c) to warming estimated by meta-analysis. Q_M, the heterogeneity caused by relevant factors (i.e., biome type, soil type, landform and precipitation seasonality), and *p* values show the statistical significance level of the difference.

**Warming level (°C)**	**Soil depth (m)**	**Sample size (*n*)**	**Biome type**		**Soil type**		**Landform**		**Precipitation seasonality**	
			Q_M	*P*	Q_M	*P*	Q_M	*P*	Q_M	*P*
**1**	0–0.3	7319	44.41	<0.01	13.37	0.27	0.2	0.91	1.66	0.44
	0.3–1	6112	19.33	0.01	9.04	0.62	2.89	0.24	14.04	<0.01
	1–2	3205	20.34	0.01	12.69	0.31	0.67	0.71	0.49	0.78
**2**	0–0.3	6090	50.43	<0.01	19.96	0.05	1.36	0.51	13.19	<0.01
	0.3–1	5076	20.53	0.01	16.4	0.13	0.84	0.66	20.55	<0.01
	1–2	2519	6.02	0.65	8.8	0.64	1.73	0.42	0.14	0.93
**3**	0–0.3	5346	66.53	<0.01	17.1	0.1	0.07	0.97	15.65	<0.01
	0.3–1	4436	11.79	0.16	13.73	0.25	2.01	0.37	25.84	<0.01
	1–2	2152	7.08	0.53	6.75	0.82	0.07	0.97	3.32	0.19
**4**	0–0.3	4765	66.49	<0.01	25.45	0.01	2.66	0.26	24.45	<0.01
	0.3–1	3885	20.12	0.01	17.08	0.11	3.76	0.15	18.07	<0.01
	1–2	1809	14.6	0.07	7.3	0.77	0.85	0.65	2.26	0.32
**5**	0–0.3	4339	78.84	<0.01	31.11	<0.01	4.16	0.13	15.45	<0.01
	0.3–1	3531	29.2	<0.01	17.17	0.1	1.41	0.49	10.95	<0.01
	1–2	1649	11.1	0.2	20.81	0.04	1.62	0.44	3.24	0.2

Supplementary Table 3. Average percentage loss of soil organic carbon (SOC) under different warming levels predicted by meta-forest model using SOC stocks from three global maps across global upland pixels. Values show the mean ± 95% confidence interval.

Data sets	Soil layers (m)	SOC stock (Pg)	Average percentage loss across global upland pixels (%)				
			+1 °C	+2 °C	+3 °C	+4 °C	+5 °C
WISE	0-0.3	830.3	-11.8±5.6	-14.2±6.3	-16.2±6.1	-19.7±6.1	-21.7±7.3
	0.3-1	996.3	-12.6±5.8	-14.9±6.4	-16.6±6.5	-19.8±6.6	-21.5±7.2
	1-2	988.2	-4.9±6.1	-5.5±6.9	-6.4±7.1	-10±6.9	-11.7±7.2
HWSD[a]	0-0.3	708.4	-7.7±6.2	-9.7±6.1	-11.6±6.2	-14.9±6.8	-16.8±7.1
	0.3-1	858.0	-4.9±5.9	-6.1±6.4	-7.4±6.3	-10.6±6.9	-12.1±7.2
SoilGrids	0-0.3	1172.2	-23.2±5.6	-27.4±6	-29.8±6.2	-33±6.3	-34.8±7
	0.3-1	1838.4	-29.8±5.5	-34.2±6.2	-36±8.5	-38.8±6.9	-40.2±7.4
	1-2	2785.5	-34.1±6.8	-38.3±6.1	-39.8±7.1	-42.1±7.4	-43.2±7.6
Global average	0-0.3[b]	903.6	-14.2±5.8	-17.1±6.1	-19.2±6.2	-22.5±6.4	-24.5±7.1
	0.3-1[b]	1181.0	-15.8±5.7	-18.4±6.3	-20±7.1	-23.1±6.8	-24.6±7.3
	1-2[c]	1886.8	-19.5±6.4	-21.9±6.5	-23.1±7.1	-26±7.2	-27.5±7.4
	0-1[b]	2134.5	-15±5.8	-17.8±6.2	-19.6±6.6	-22.8±6.6	-24.5±7.2
	0-2[c]	4305.5	-19.4±5.9	-22.4±6.3	-24.2±6.9	-27.2±6.7	-28.9±7.3

[a], HWSD does not report SOC stock in the 1–2 m soil layer depth.
[b], the average values were derived based on SOC stock estimates of WISE, HWSD and SoilGrids.
[c], the average values were derived based on SOC stock estimates of WISE and SoilGrids

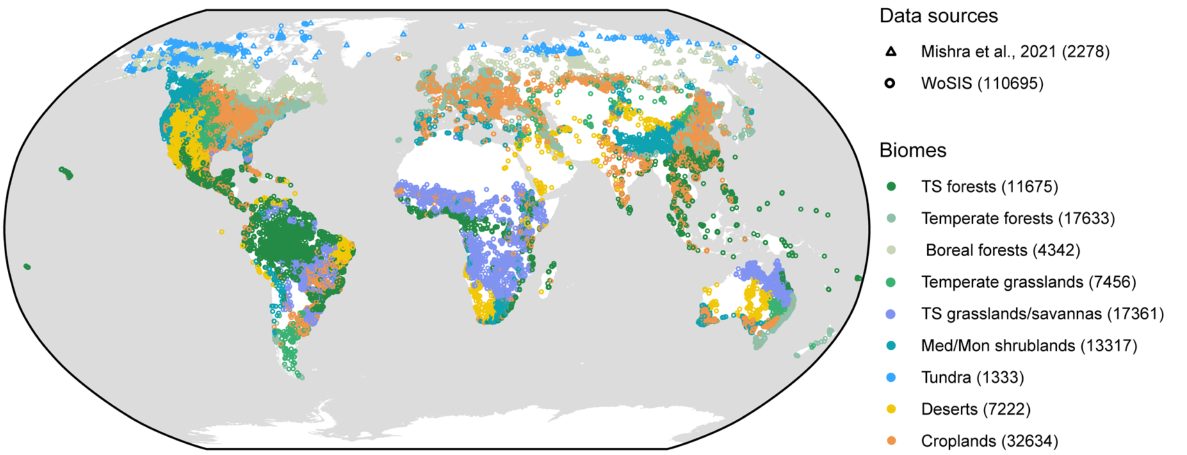

Supplementary Fig. 1. Data sources and the location of soil profiles used in this study. Numbers in parentheses are the number of soil profiles. TS, tropical/subtropical; Med/Mon, Mediterranean/montane.

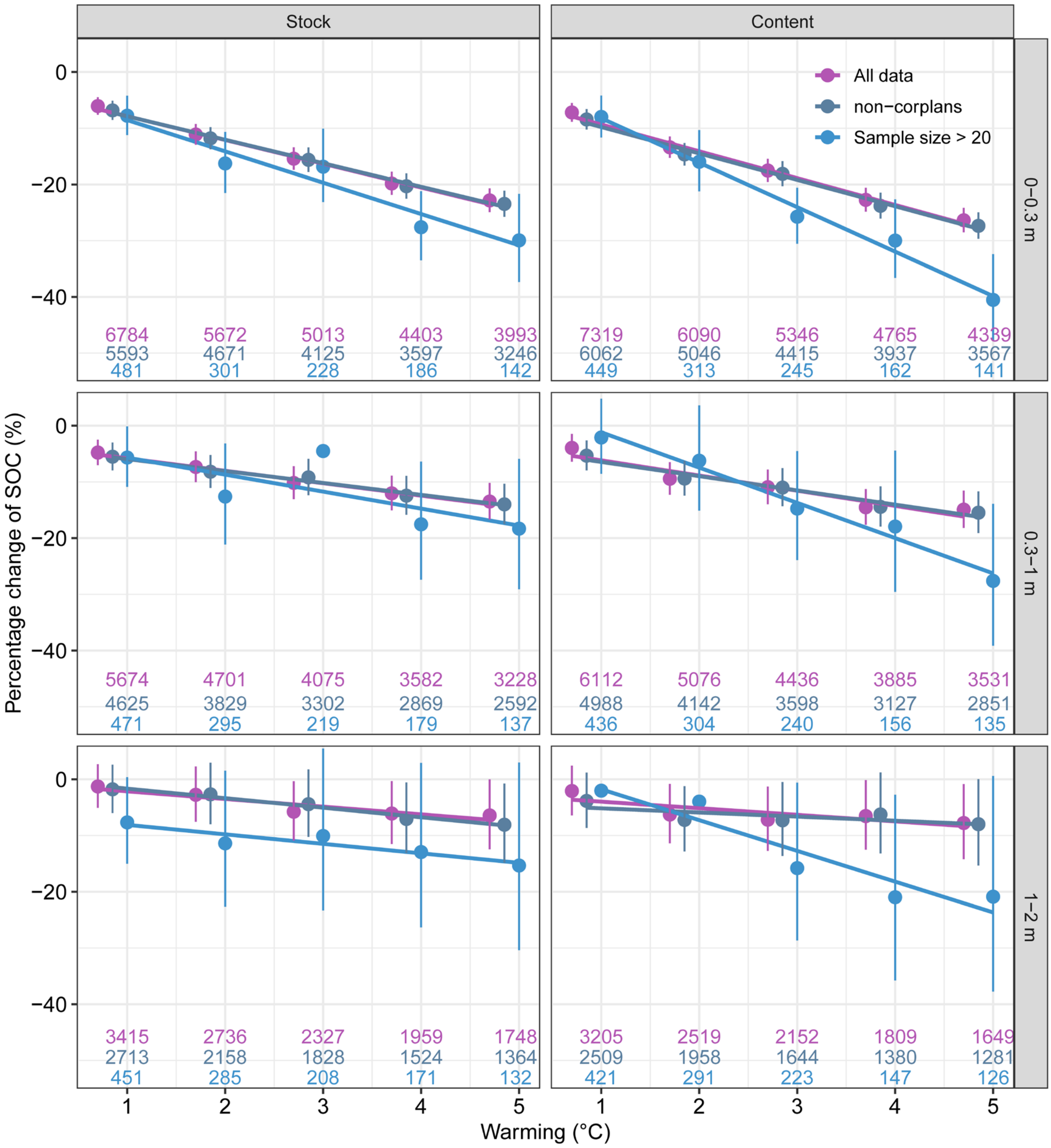

Supplementary Fig. 2. Sensitivity assessment of global percentage response of soil organic carbon (SOC) stock and content to warming. Non-croplands, excluding soil profiles from croplands; sample size > 20, excluding groups with less than 20 soil profiles. Sample size (i.e., the number of soil profiles used to estimate the response) is shown for each soil layer depth (i.e., 0–0.3, 0.3–1 and 1–2 m) under each warming level (i.e., 1, 2, 3, 4, 5 °C warming). Vertical bars show the 95% confidence interval. Solid lines show the linear regression between response and warming level in three soil layers depths. Values for warming levels are jittered to make the points more distinct.

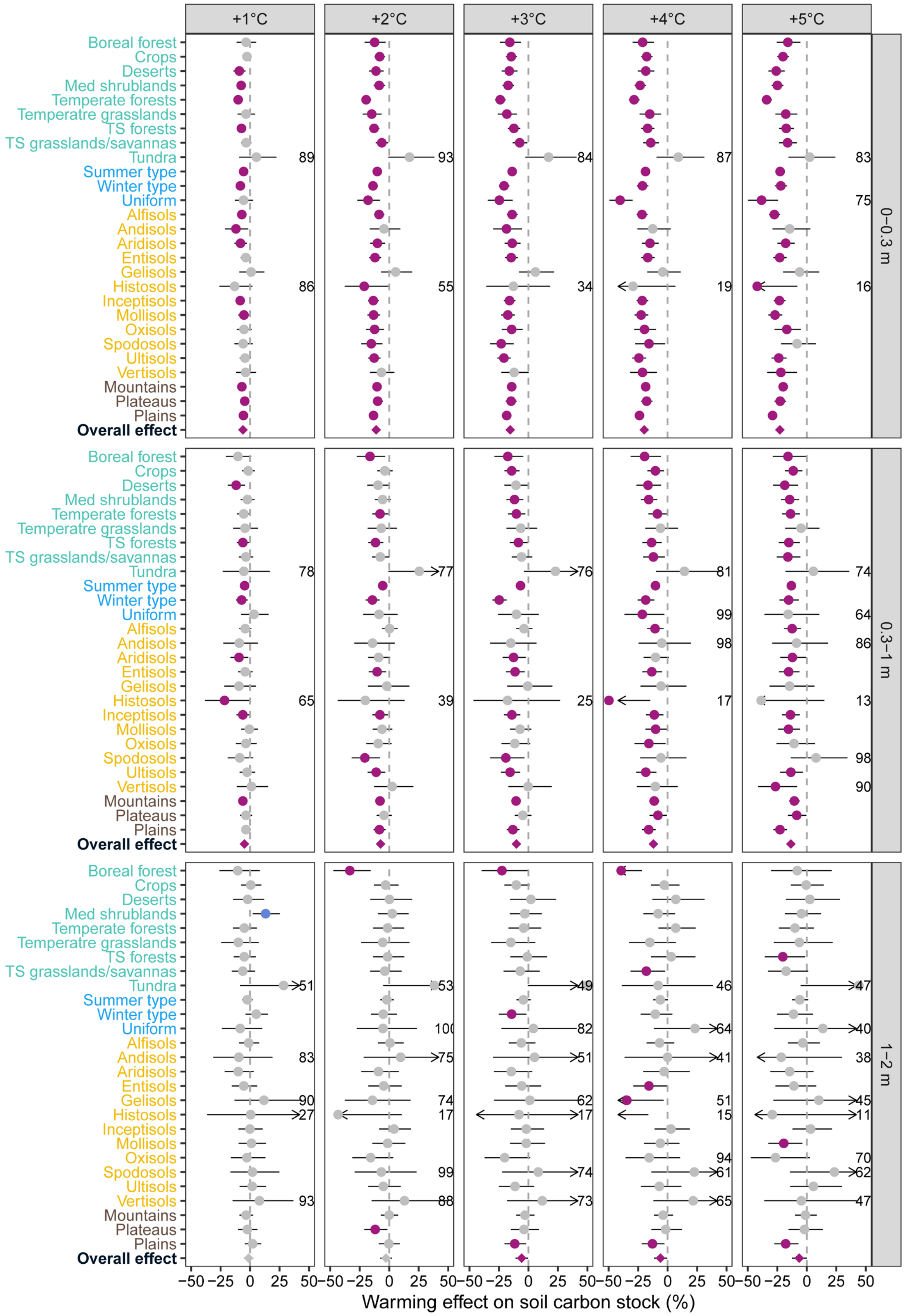

Supplementary Fig. 3. Response of soil organic carbon (SOC) stock to warming under different environmental conditions. From top to bottom panels, three soil layer depths (i.e.,

0–0.3, 0.3–1 and 1–2 m); from left to right panels, five warming levels (i.e., 1, 2, 3, 4, 5 °C). Four groups of environmental conditions are considered: biome type (green colour y-axis labels), precipitation seasonality (blue), soil order (gold), and landform (brown). Overall effect shows the global estimates. Dots with bars show the mean effects with 95% confidence intervals (CI). Purple and blue dots indicate that the effects are significantly (i.e., the 95% CI does not overlap with zero) negative and positive, respectively; while grey dots indicate that the effects are insignificant. Arrows indicate that 95% CI extends beyond the limits of x-axis. If a dot under a specific environment is missed, the effect size is located beyond the limits of x-axis. Values show sample sizes of the environment where sample size is <100. TS, tropical/subtropical; Med/Mon, Mediterranean/montane. See more details about the environmental variables in the Method.

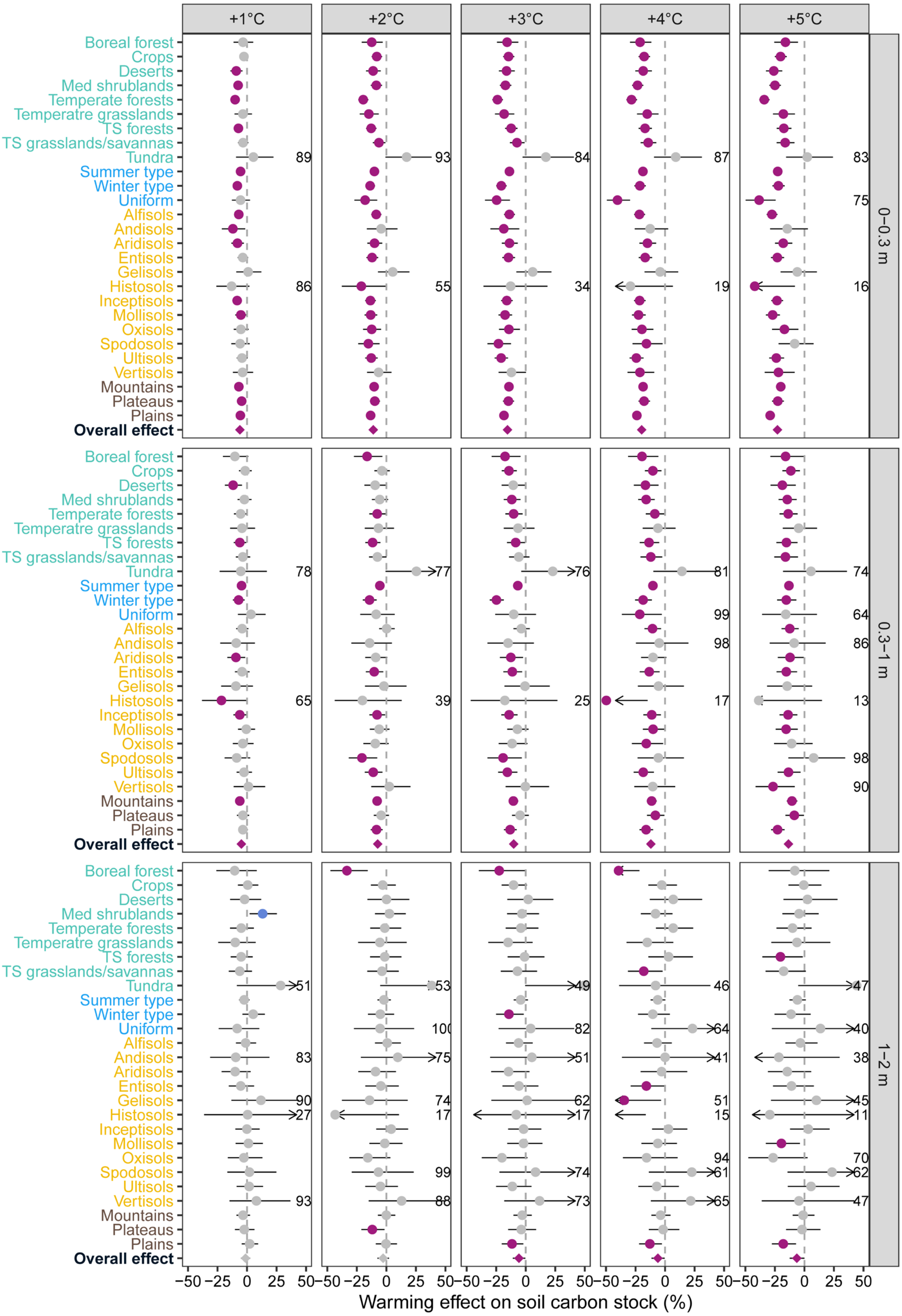

Supplementary Fig. 4. Response of soil organic carbon (SOC) content to warming under different environmental conditions. From top to bottom panels, three soil layer depths (i.e., 0–0.3, 0.3–1 and 1–2 m); from left to right panels, five warming levels (i.e., 1, 2, 3, 4, 5 °C).

Four groups of environmental conditions are considered: biome type (green colour y-axis labels), precipitation seasonality (blue), soil order (gold), and landform (brown). Overall effect shows the global estimates showed in Fig. 1 in the main text. Dots with bars show the mean effects with 95% confidence intervals (CI). Purple and blue dots indicate that the effects are significantly (i.e., the 95% CI does not overlap with zero) negative and positive, respectively; while grey dots indicate that the effects are insignificant. Arrows indicate that 95% CI extends beyond the limits of x-axis. If a dot under a specific environment is missed, the effect size is located beyond the limits of x-axis. Values show sample sizes of the environment where sample size is <100. TS, tropical/subtropical; Med/Mon, Mediterranean/montane.

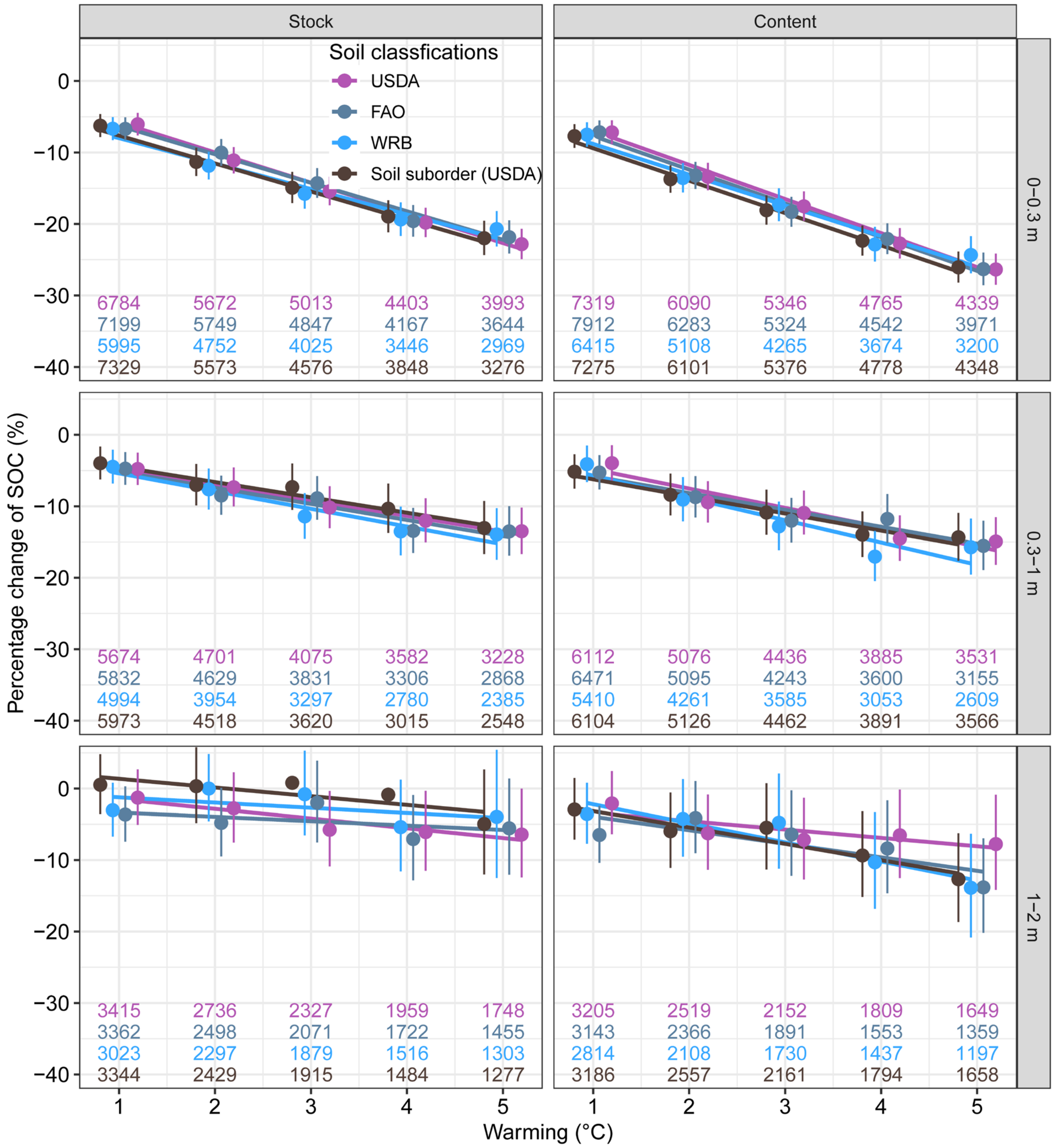

Supplementary Fig. 5. The effects of soil types on global percentage response of soil carbon stock and content to warming. Sample size (i.e., the number of soil profiles used to estimate the response) is shown for each soil layer depth (i.e., 0–0.3, 0.3–1 and 1–2 m) under each warming level (i.e., 1, 2, 3, 4, 5 °C warming). Vertical bars show the 95% confidence interval. Solid lines show the linear regression between response and warming level in three soil layers depths. Values for warming levels are jittered to make the points more distinct.

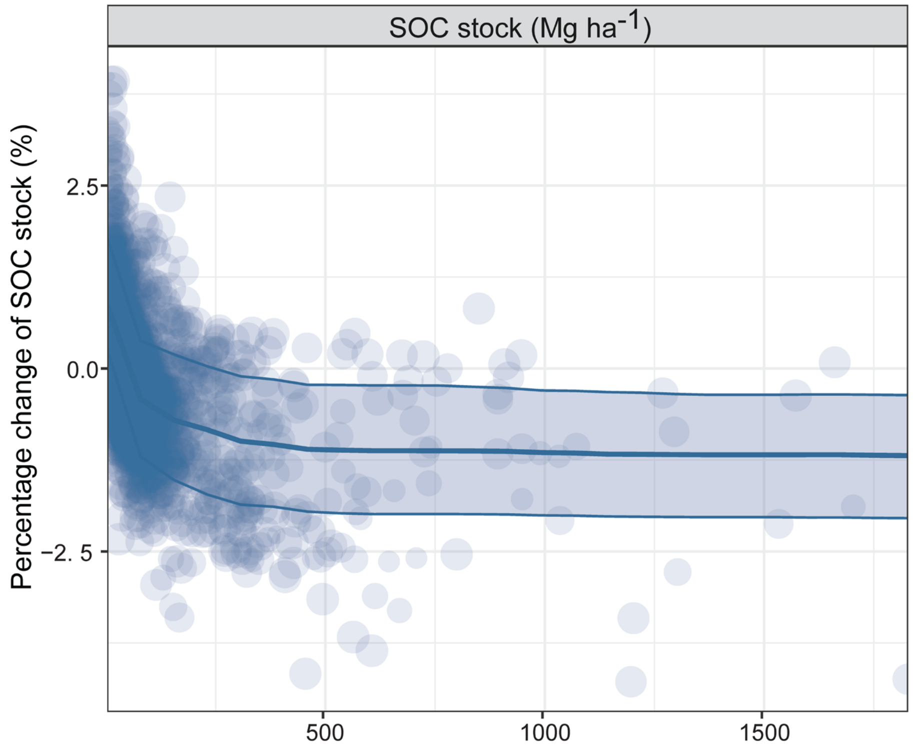

Supplementary Fig. 6. Partial dependence of percentage SOC stock changes under 2 °C warming on existing SOC stock. Partial dependence depicts the marginal effect of a variable on the response after taking into account the effects of other variables in the model. The shade area represents the 95% confidence interval of the regression line.

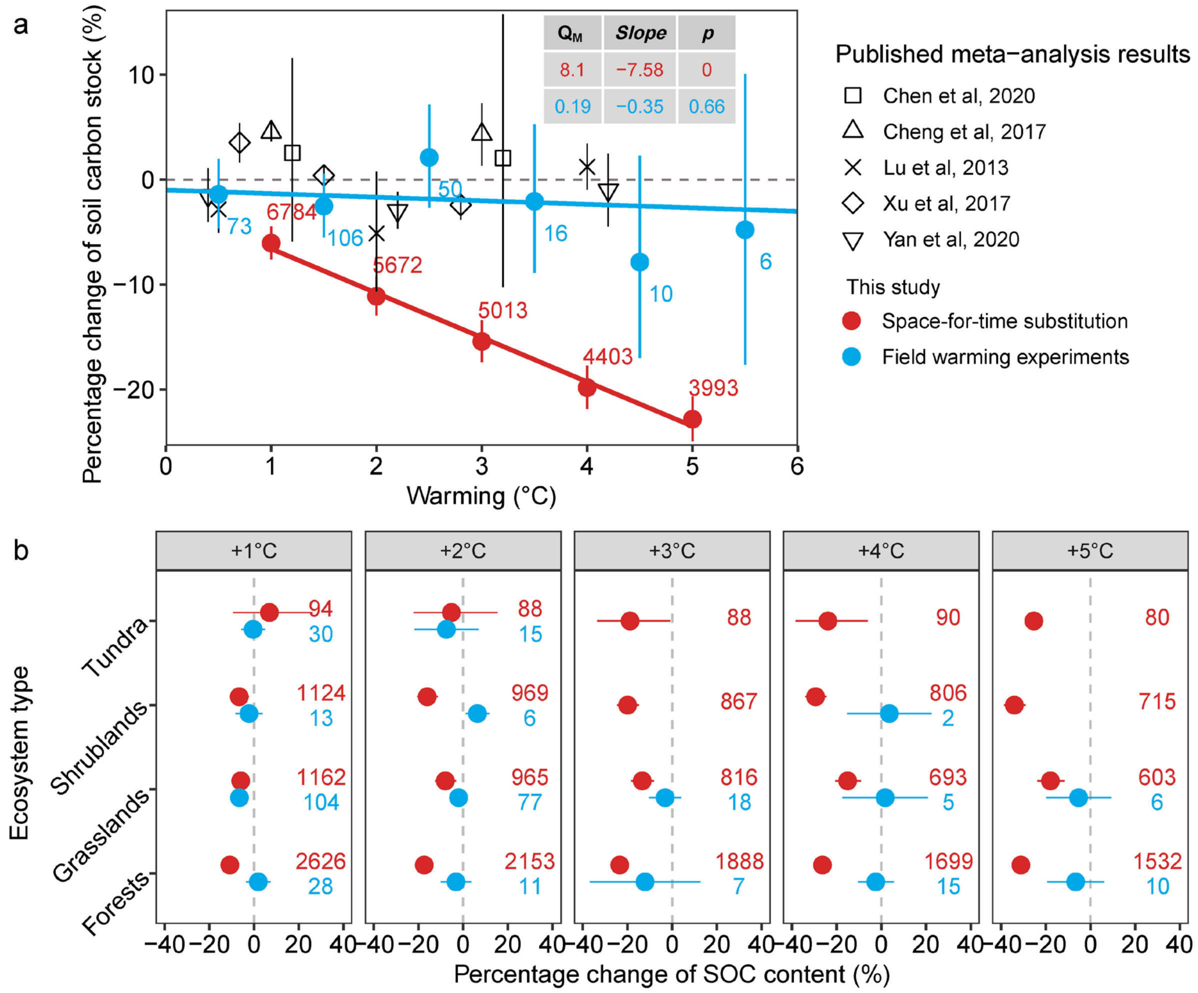

Supplementary Fig. 7. Comparison of estimated soil organic carbon (SOC) changes as impacted by warming with field warming experiments. a, response of SOC to warming estimated by different approaches. Most field warming experiments limit SOC measurements to the top 0.2 m soil; the estimates in this study presented are the response in the top 0–0.3 m soil layer. Numbers below or besides the symbols are sample sizes. The color lines are the regression line fitted by weighted meta-regression. Inset table shows the statistics of the regression including the determination coefficient (R^2), regression slope and *p* value. **b**, ecosystem-specific comparison with estimates in this study. Biomes are grouped into tundra, shrublands, grasslands, and forests. In both **a** and **b**, dots with bars show the mean effect sizes with 95% confidence intervals.

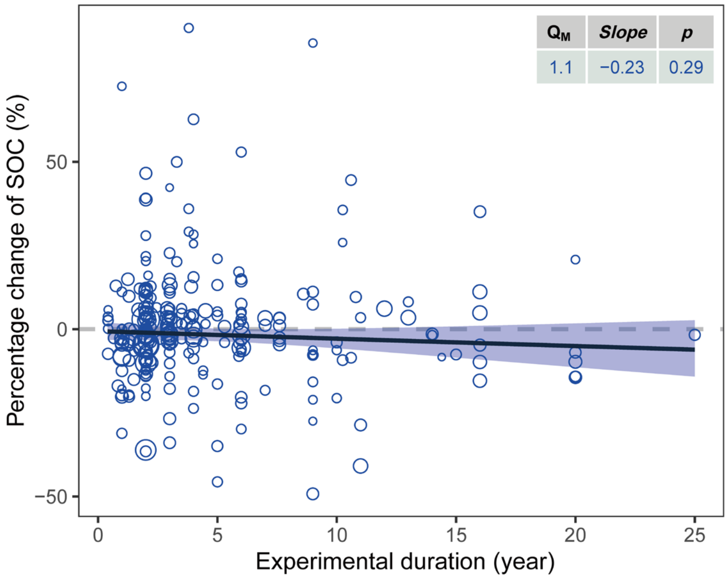

Supplementary Fig. 8. The relationship of the response with experimental duration. The line is the regression line fitted by weighted (circle size indicates the weight) meta-regression, and the shade area represents the 95% confidence interval.

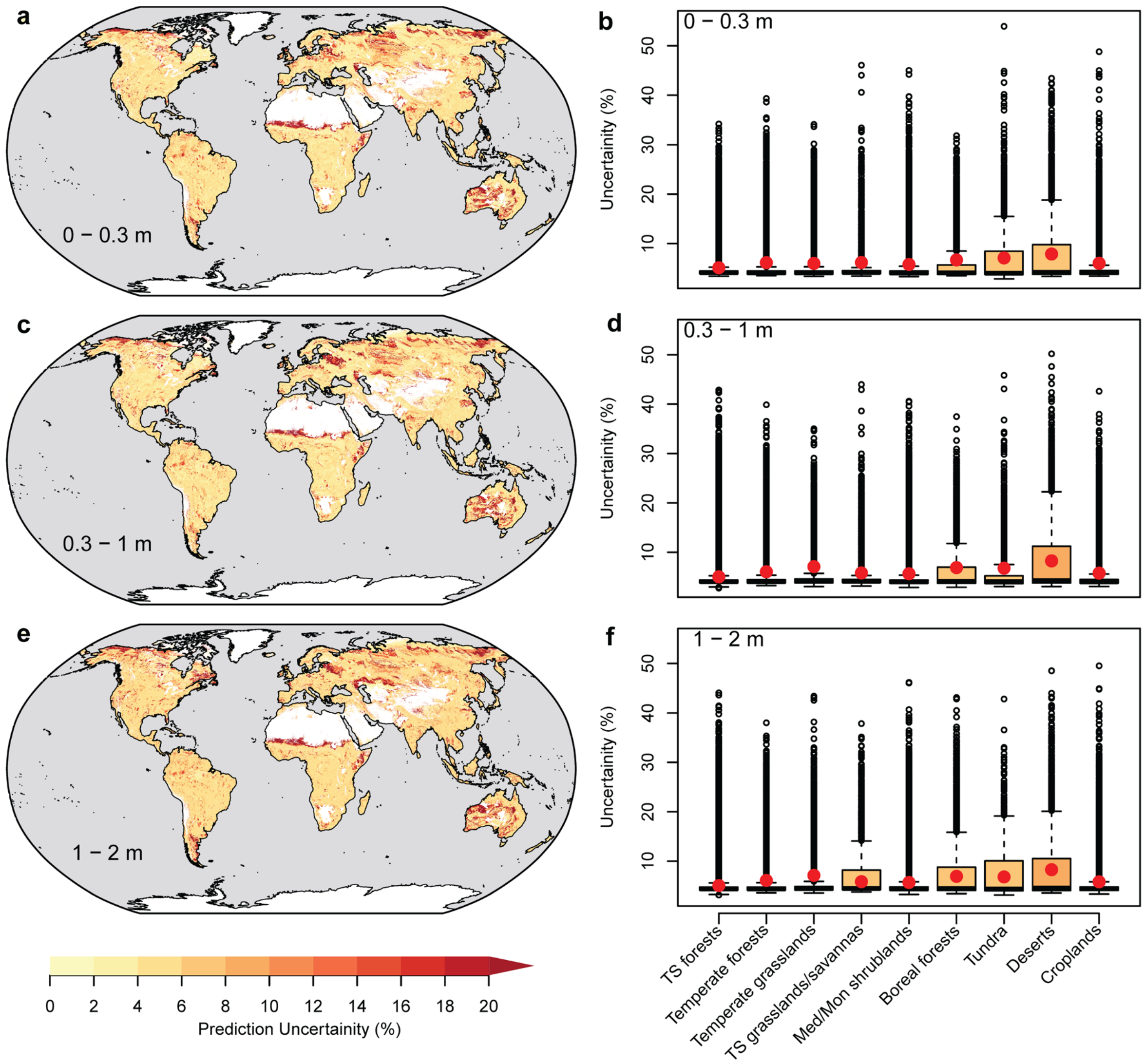

Supplementary Fig. 9. Uncertainty of percentage changes of soil organic carbon (SOC) stocks under 2°C warming across the globe. Top, middle, and bottom panels: three soil layer depths (i.e., 0–0.3, 0.3–1, 1–2 m) respectively. Left panels: maps of the spatial distribution of the changes; right panels, aggregated by biome types. The maps are produced by applying machine learning-based meta-forest models constrained by the global soil profiles across the globe at the resolution of 1 km. Prediction uncertainty is presented as one standard deviation of estimates of meta-forest trees in each 1 km pixel. TS, tropical/subtropical; Med/Mon, Mediterranean/montane.

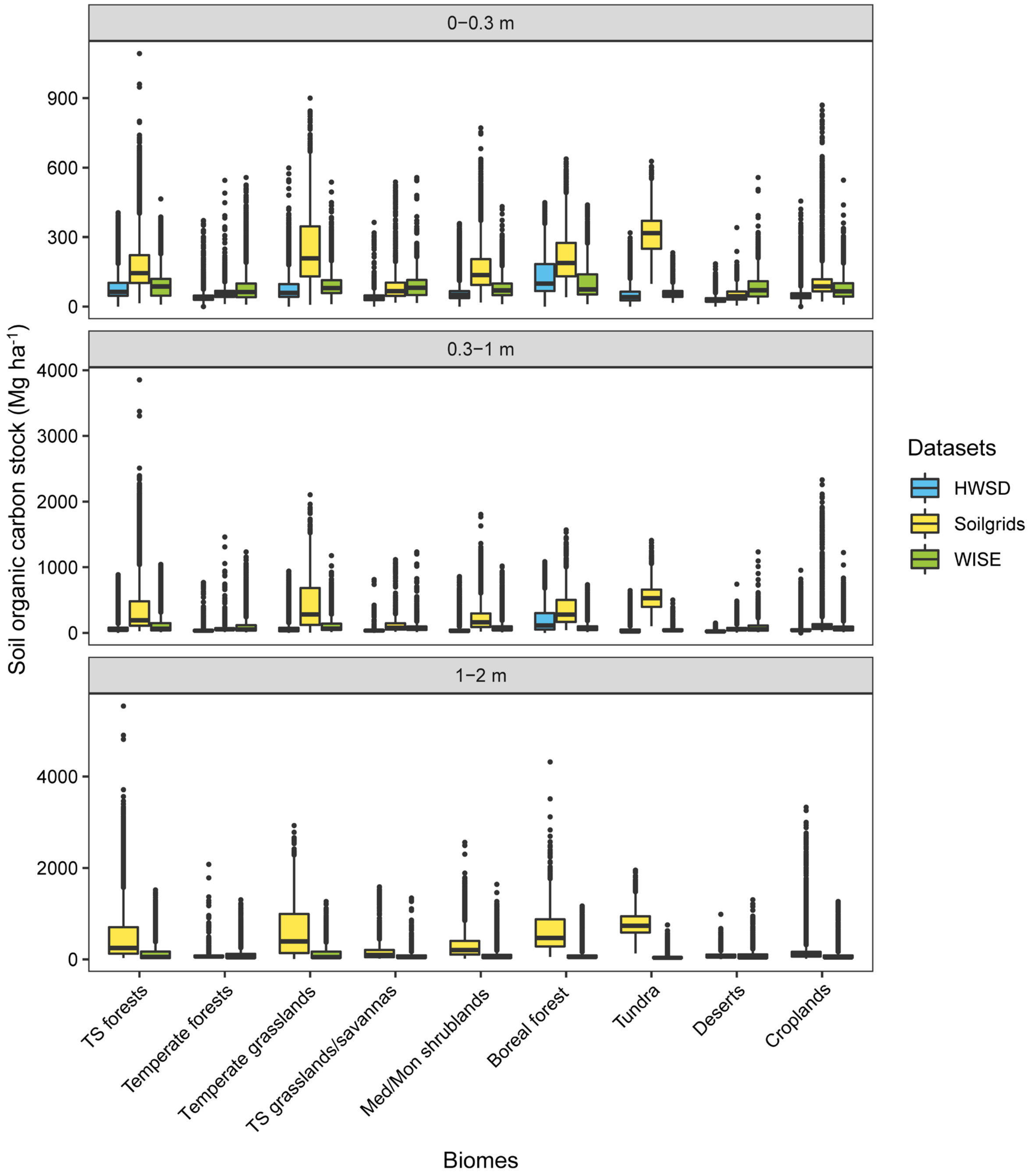

Supplementary Fig. 10. Soil organic carbon estimated by HWSD, SoilGrids and WISE in nine biomes. Top, middle, and bottom panels show the results for three soil layer depths (i.e., 0–0.3, 0.3–1, 1–2 m), respectively. HWSD does not include SOC estimates in the 1–2 m soil depth. TS, tropical/subtropical; Med/Mon, Mediterranean/montane.

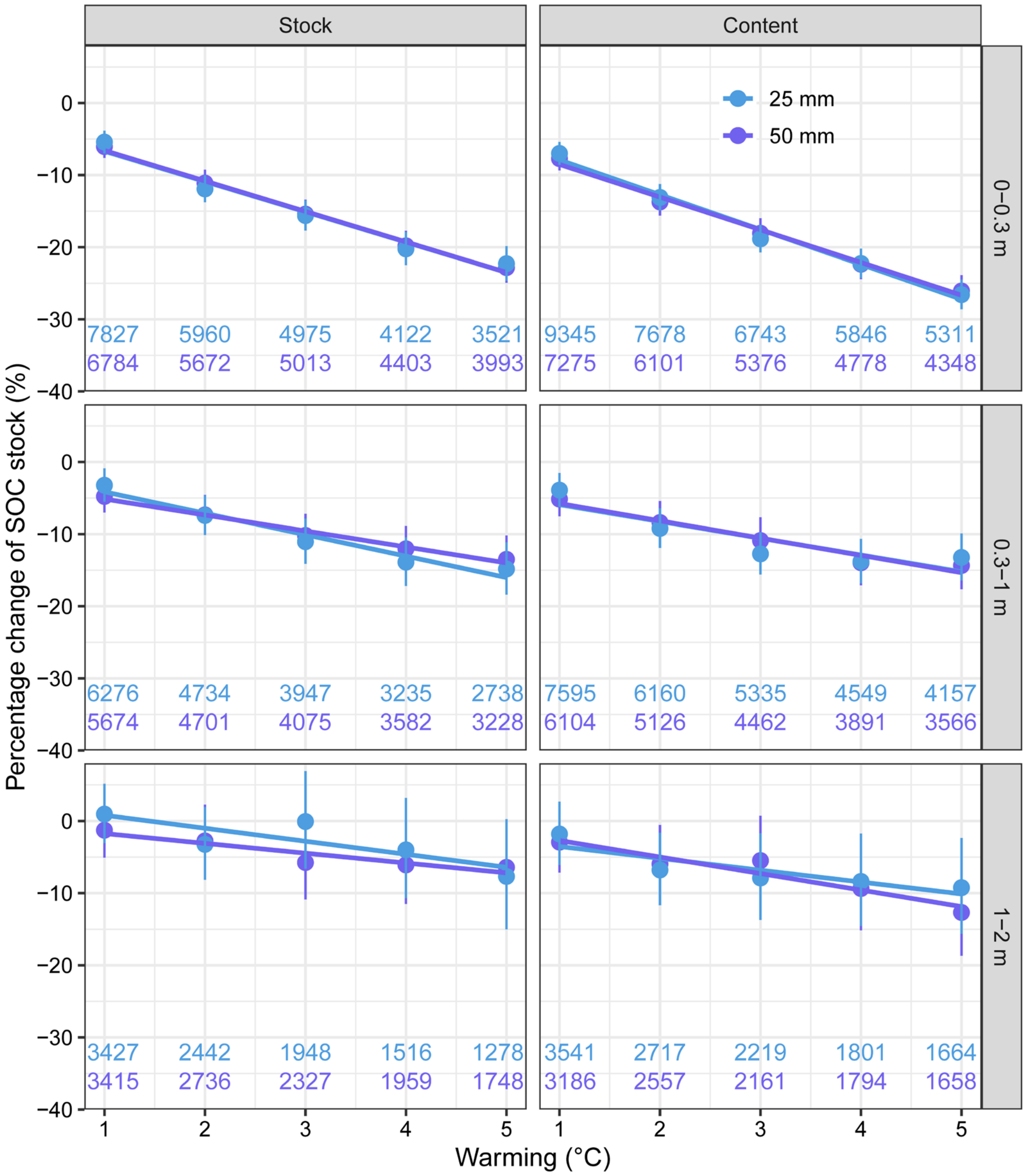

Supplementary Fig. 11. Responses of soil organic carbon (SOC) to warming as impacted by precipitation deviation. (**a**) SOC stock, (**b**) SOC content. Precipitation deviation is defined as the absolute difference of precipitation between "ambient" and "warm" soils. Sample size (i.e., the number of soil profiles used to estimate the response) is shown for each soil layer depth (i.e., 0–0.3, 0.3–1 and 1–2 m) under each warming level (i.e., 1, 2, 3, 4, 5 °C warming). Vertical bars show the 95% confidence interval. Solid lines show the linear regression between response and warming level in three soil layers depths. Values for warming levels are jittered to make the points more distinct.
