# Supplementary material for: Global soil profiles indicate depth-dependent soil carbon losses under a warmer climate
Source: Nat Commun. 2022 Sep 20;13:5514. doi: 10.1038/s41467-022-33278-w (PMC9489695; doi:10.1038/s41467-022-33278-w)
Supplement: Supplementary file 3 — Description of Additional Supplementary Files [file 41467_2022_33278_MOESM3_ESM.pdf]

Description of Additional Supplementary Files

File Name: Supplementary Data 1
Description: Data from field warming experiments and references used to collect the data.
